# Case of Premature Parturition Artificially Excited

**Published:** 1817-01

**Authors:** James Gibbon

**Affiliations:** Member of the Royal College of Surgeons.


### *For the London Medical and Physical Journal.*

*Case of Premature Parturition artificially excited;* by JAMES GIBBON, Esq. Member of the Royal College of Surgeons.

IN the month of August 1812, I attended Mrs. D. of this town, in a case of very protracted and laborious labour, of her first child, which was still-born, and had apparently been dead about two days. Great uterine hæmorrhage followed the separation of the placenta, and she was reduced almost to the last extremity. It was several months, and with the greatest attention, before she recovered her usual state of health. She was considerably advanced in years, and of a rigid fibre. There was no deficiency of pains throughout the whole of this lingering labour. Under these circumstances, I thought it right to interfere but little with the operations of Nature. The occasional administration of injections, and the introduction of the catheter, were required. Although I was aware, during the progress of this labour, that the capacity of the pelvis was small, I did not suppose the contraction of its brim to be so great as I afterwards found it. The head of the child was so softened, by having laid long in the pelvis after its death, as to prevent my forming an accurate opinion of the degree of pressure it had borne in passing through the upper aperture.

She became pregnant again, and suffered an early miscarriage without any alarming symptoms, in the month of August 1813.

A third time she became pregnant, and, after a still more lingering and laborious labour, was again delivered of a dead child in January 1815; most happily, no hæmorrhage followed. I now ascertained that the brim of the pelvis was much contracted, and ventured to predict that it was highly improbable she would ever bring forth a mature living child.

A fourth state of pregnancy ensued, and she expected to fall into labour about the middle of December 1816. She was anxious in the extreme to bring forth a living child. With a view of securing her own safety, and enabling her to do this, I proposed exciting a premature labour in the beginning of the last month.—She acceded to it. After the bowels were well emptied, I proceeded to attempt a moderate dilatation of the os tincæ. I met with considerable difficulty in effecting this, owing to the smallness of the canal of the pelvis, and the uterus resting upon the protruding part of the sacrum. After many cautious attempts, I succeeded, however, however, on Thursday evening, the 7th of December, in procuring a discharge of the fœtal waters; slow natural labour soon followed. That night she had some sleep, though occasionally disturbed with regular pains. The child was then lively, and I had reason to believe, from my repeated examinations, that the head presented favourably. During the whole of Friday, the 8th, she continued in slow labour, and had but little sleep during the night. On Saturday, the 9th, my patient became uneasy, from anticipating sufferings equally dreadful and protracted as those she had felt during her former labours. Early in the evening of that day, I gave her a full dose of opium in a cordial draught, with the happy effect of procuring for her some very refreshing sleep. From this she awoke with strong pains. Early on Sunday morning, the child rapidly descended into the pelvis, and, to my great surprize, exhibited a breech presentation. Even at this period of the labour, I feel assured that the child was quite alive; but, when its head came in contact with the os sacrum, although the pains were extremely strong, the progress of the labour was much retarded. I need not say to an experienced accoucheur, that such unavoidable delay in a breech presentation was necessarily fatal to the child.

In much less time, however, than I anticipated, she was safely delivered of a third dead child, and rapidly recovered without a single untoward symptom.

It appeared that the head of the child passed through the superior aperture of the pelvis in the most unfavourable position, for the parietal bone of one side was so strongly forced against the protruding part of the os sacrum, by the violence of the pains, as to occasion a very deep indentation upon it. This clearly accounted for the delay and consequent death of the child.

I am now of opinion, that, if the labour had been brought on two or three weeks sooner, the chances would have been greater in favour of a living child. From the great contraction, however, of the pelvis in this case, it is not probable that a child of seven months can be born alive, except it is a natural presentation. It is possible, I fear, that the deformity of the bones of the pelvis is here increasing.

This case may be considered as an additional instance of the safety and advantage of exciting premature labour, in those subjects who have the misfortune to bear children with a contracted pelvis; my patient declaring that she suffered incalculably less in this, than in her former confinements.

*Bedford; December* 9, 1816.

